# Older adults compensate for switch, but not mixing costs, relative to younger adults on an intrinsically cued task switching experiment

**DOI:** 10.3389/fnagi.2023.1152582

**Published:** 2023-04-20

**Authors:** Teal S. Eich, Christopher Langfield, Jayant Sakhardande, Yunglin Gazes, Christian Habeck, Yaakov Stern

**Affiliations:** ^1^Leonard Davis School of Gerontology, University of Southern California, Los Angeles, CA, United States; ^2^Cognitive Neuroscience Division and The Taub Institute, Department of Neurology, Columbia University Medical Center, New York City, NY, United States

**Keywords:** task switching, costs, executive processes, compensation, working memory, aging

## Abstract

**Introduction:**

Aging negatively impacts the ability to rapidly and successfully switch between two or more tasks that have different rules or objectives. However, previous work has shown that the context impacts the extent of this age-related impairment: while there is relative age-related invariance when participants must rapidly switch back and forth between two simple tasks (often called “switch costs”), age-related differences emerge when the contexts changes from one in which only one task must be performed to one in which multiple tasks must be performed, but a trial-level switch is not required (e.g., task repeat trials within dual task blocks, often called “mixing costs”). Here, we explored these two kinds of costs behaviorally, and also investigated the neural correlates of these effects.

**Methods:**

Seventy-one younger adults and 175 older adults completed a task-switching experiment while they underwent fMRI brain imaging. We investigated the impact of age on behavioral performance and neural activity considering two types of potential costs: switch costs (dual-task switch trials minus dual-task non-switch trials), and mixing costs (dual-task non-switch minus single-task trials).

**Results:**

We replicated previous behavioral findings, with greater age associated with mixing, but not switch costs. Neurally, we found age-related compensatory activations for switch costs in the dorsal lateral prefrontal cortex, pars opercularis, superior temporal gyrus, and the posterior and anterior cingulate, but age-related under recruitment for mixing costs in fronto-parietal areas including the supramarginal gyrus and pre and supplemental motor areas.

**Discussion:**

These results suggest an age-based dissociation between executive components that contribute to task switching.

## 1. Introduction

From making breakfast to driving home from work, the execution of most activities in daily life requires the ability to switch between different tasks. A large literature surrounding the cognitive and neural mechanisms that contribute to task switching abilities has demonstrated that switching between two relatively simple tasks as opposed to repeating the same task produces significant declines in behavioral performance, negatively affecting both response speed and accuracy (Allport et al., 1994; Rogers and Monsell, 1995; Allport and Wylie, 1999; Kiesel et al., 2010), as well as increasing blood oxygen level-dependent brain activity in a network of prefrontal, fronto-parietal, and striatal regions (Dove et al., 2000; Sohn et al., 2000; Smith et al., 2001; Brass and von Cramon, 2002; Braver et al., 2003; Sylvester et al., 2003; Aron et al., 2004; Badre and Wagner, 2006). Given the significant changes to both executive functioning and neural systems that accompany healthy aging (West, 1996; Cabeza, 2002; Verhaeghen and Cerella, 2002; Salat et al., 2004; Botvinick, 2008; Cabeza and Dennis, 2012), it is not surprising that task switching effects have been shown to be augmented in old age both behaviorally (Wasylyshyn et al., 2011) and neurally (Botwinick et al., 1958; Kramer et al., 1999; DiGirolamo et al., 2001; Smith et al., 2001; Shallice et al., 2008; Gold et al., 2010; Jimura and Braver, 2010).

The costs associated with switching between tasks can be measured in different ways, however, and these different indexes may reflect cognitive processes that are differentially affected in aging. A number of fMRI investigations including those from our own group (Gazes et al., 2012; Oh et al., 2016) have focused upon “dual-task” (Koch et al., 2018) or “global” (Braver et al., 2003) costs, in which performance across entire blocks in which the participant has to make only one type of decision (e.g., single-task blocks which contain only task-repeat trials, e.g., *AAAAA*) are contrasted with performance on blocks of trials in which the participant must switch between two different tasks. Task switches in these dual-task blocks are typically randomized rather than being sequential, and thus these blocks contain both task-switch and task-repeat trials (e.g., *AAABAB*; Jersild, 1927). However, performance and brain activity is investigated collapsing across these different types of trials. Such comparisons typically show a large age-related effect: while younger adults perform worse in the dual-task blocks compared to the single task blocks, this dual-task cost is substantially larger for older adults (Wasylyshyn et al., 2011).

Measuring switch costs across a block of trials, particularly one that contains both switch and no-switch trials, confounds multiple cognitive processes that are known to be differentially affected in aging, including working memory (Nyberg et al., 2012), attentional selection or priming (D'Angelo et al., 2016), inhibition (Hasher et al., 2007; Eich et al., 2018), and conflict resolution and stimulus–response congruency effects (Ward, 1982; Rogers and Monsell, 1995; Meiran, 1996; Eich et al., 2016a). Because of this, it is difficult to tease apart the precise cognitive components that may contribute to age-related differences in task-switching. Calculating costs based upon local, trial-level differences using a trial-based MRI design (Rosen et al., 1998; Huettel, 2012), on the other hand, may provide for a more precise operationalization of cognitive mechanisms involved in task switching and help clarify when and why they are affected in aging (Gajewski et al., 2018).

Two main types of costs can be calculated based on trial level data. The first, typically referred to as a “local switch” cost, compares performance for switch trials that occur in a dual-task block (e.g., *AABBA*) to task repeat trials that occur in a dual-task block (e.g., *AABBA*). Thus, only data from the dual-task blocks are considered, and thus the context of being in a dual task block is held constant. According to Braver (2012), the cognitive processes associated with local switch costs reflect a late, reactive strategy that depends on information encountered directly from the stimuli. This processing, thus, occurs in a bottom-up manner. fMRI studies have associated these transient shifts from one task to another with activation in lateral areas in the PFC, including Broadman Area (BA) 9, 46, 44, and 45, along with the inferior frontal gyrus in younger adults (Brass et al., 2005; Montojo and Courtney, 2008; Jimura and Braver, 2010; Nee and Brown, 2013; Richter and Yeung, 2014; Muhle-Karbe et al., 2016), areas often associated with the ability to resolve interference from conflicting information (Eich et al., 2014; Eich et al., 2017; Eich et al., 2021). When local switch costs were compared for younger and older adults in a meta-analysis of 26 published articles (with 36 independent participant groups), no specific age deficits were found behaviorally (Wasylyshyn et al., 2011). Recently, Nashiro et al. (2018), who investigated age-related differences in global and local switch costs (but not mixing costs), as well as congruency effects, reported that activations in right precentral and postcentral gyri were associated with local switch costs. However, they did not find age-related differences behaviorally, nor did they find neural differences in activation in this area in response to local switch costs between younger and older adults (Nashiro et al., 2018).

The second type of trial-level cost that can be investigated is called a “mixing” cost [or sometimes “global selection cost” (Keele and Rafal, 2000; Mayr, 2001; Steinhauser and Hubner, 2005)]. Mixing costs are indexed by comparing performance across two repeated (non-switch) tasks in a dual-task block (e.g., *AABBA*) to single-task block trials (which by definition are *all* non-switch trials, e.g., *AAAAA*). Whereas local switch costs are thought to reflect transient, item-specific cognitive control processes needed on a trial-by-trial basis to resolve interference from the alternative task, reconfigure the new task-set by upregulating attentional resources toward the currently relevant task-set, and adopt the correct stimulus–response mapping (Monsell, 1978; Kray and Lindenberger, 2000; Monsell, 2003; Monsell Sumner and Waters, 2003; Jonides and Nee, 2005, 2006; Philipp et al., 2008; Kiesel et al., 2010), mixing costs, on the other hand, have been associated with “sustained” cognitive processes. Although task-repeat trials within a dual-task block are identical to single-task trials (that is, the stimulus–response mappings and task rules do not need to be updated across these dyads), ongoing attentional resources are needed to consistently monitor for task changes in dual-task as opposed to single-task blocks, to manage competition between different tasks through either inhibitory or activation processes (Meiran et al., 2000; Rubin and Meiran, 2005), and to keep one vs. multiple task-sets in working memory over time (Fagot, 1994; Los, 1996; Kray and Lindenberger, 2000; Meiran, 2000; Meiran et al., 2000; Pashler, 2000; Meiran and Gotler, 2001); c.f. (Rubin and Meiran, 2005). According to Koch et al. (2005, 2018), these last two processes—inhibition and working memory—are of particular import to performance reductions on this measure because “the competing task set is still lingering in working memory and cannot be fully de-activated because it will be needed soon again.” In several studies that have investigated mixing costs behaviorally, age-related effects have been reported, and, when the magnitude of effects were directly compared to those of local switch costs, mixing costs were reported to be significantly larger than local switch costs (Kray and Lindenberger, 2000; Mayr, 2001; Meiran et al., 2001).

A number of studies have used fMRI to investigate brain activity associated with global switch costs (Braver et al., 2003; Jimura and Braver, 2010; Nashiro et al., 2018), which may be associated with early, proactive, top-down modulations of ongoing processes (Braver and Barch, 2002). These studies report activation peaks in the anterior PFC including BA 10, along with areas 6, 8, and 32 in the anterior portion of the anterior cingulate cortex in younger adults. In older adults, increased activity in typical task switching related fronto-parietal regions is associated with a greater cost (worse performance). However, while both global switch and mixing costs reflect the cost of being in the context in which one might have to shift between task-sets, only the mixing-cost reflects this context free from executive processes needed to actually carry out the switch. Thus, while interactions between local and mixing costs and age have been previously explored behaviorally—with dissociable behavioral effects reported in old age—and local and global costs have been examined using fMRI—with age-effects found for global costs, to our knowledge, there have been no published reports using fMRI to investigate the neural basis of both local switch and mixing costs in older and younger adults.

The primary goal of the current study was to therefore assess how aging affects patterns of brain activation in response to trial-level executive control demands as measured by local switch costs and mixing costs. To this end, participants in our study completed a task switching task while undergoing fMRI. We assessed both behavioral performance across these two types of costs, and then explored the brain areas activated in response to each cost within each age group, and across them, allowing us to identify areas that were over recruited (potentially representing compensatory activation) or under-recruited (potentially representing functional deficits). In so doing, we hoped to provide a clearer understanding of the cognitive and neural mechanisms that underlie task switching performance costs, and the changes that occur to them as a function of age.

## 2. Materials and methods

### 2.1. Participants

Participants were recruited to the study using a market mailing procedure that targeted individuals living within 10 miles of the Columbia University Medical Center. Individuals who had current neurological or psychiatric diagnoses or dementia, defined as a score less than or equal to 135 on the Mattis Dementia Rating Scale (Mattis, 1988), were excluded prior to being tested. Informed consent was approved by the Internal Review Board of the College of Physicians and Surgeons of Columbia University, and was obtained for all participants prior to study participation. Data were collected from 254 participants, including 72 younger adults (age range 20–31, *M* age = 26.11, 66% female, *M* years education = 15.63) and 182 older adults (age range 60–71, *M* age = 65.31, 54% female, *M* years education = 16.02). Eight participants’ imaging data were unusable, leaving a final sample of 246 participants, including 71 younger adults (age range 20–31, *M* age = 26.10, 65% female, *M* years education =15.62) and 175 older adults (age range 60–71, *M* age = 65.34, 54% female, *M* years education =15.97).

### 2.2. Stimuli

The stimuli consisted of 12 letter-stimuli, presented in either red, green or white, pseudorandomly chosen from the set (A, E, I, O, a, e, I, o, C, G, K, P, c, g, k, and p). Task stimuli were back-projected onto a screen located at the foot of the MRI bed using an LCD projector. Participants viewed the screen *via* a mirror system located in the head coil and, if needed, had vision corrected to normal using MR compatible glasses (manufactured by SafeVision, LLC. Webster Groves, MO).

### 2.3. Experimental protocol

**Figure 1:**
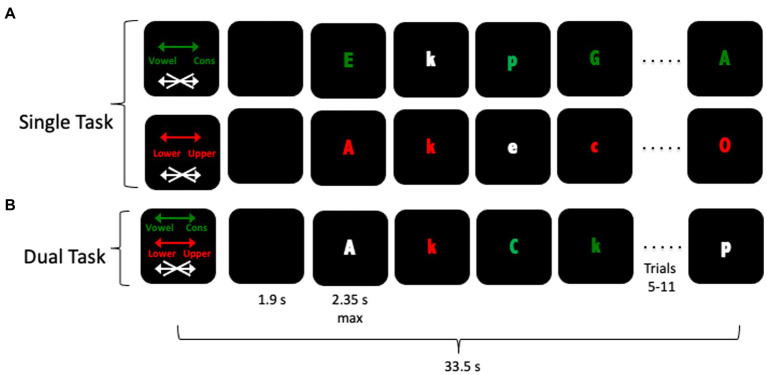
Schematic of the fMRI task. Participants received an instruction cue followed by 12 trials in which they used their left and right index fingers to indicate their response. The color of the letter indicated which task—Vowel/Consonant (green) or Upper/Lower (red) cased or NoGo (white)—should be performed. Panel **(A)** represents both types of single-task blocks, in which only one of the two tasks sets were cued. Panel **(B)** represents a dual-task block, in which participants alternated between the two different tasks.

As described previously (Eich et al., 2016b), participants received 24 33.5 s blocks, broken into 4 fMRI runs, of an intrinsically cued task switching paradigm based on Koechlin et al. (2003). The different block types, illustrated in Figure 1, each included an instruction cue presented for 2.8 s, followed by a blank screen for 1.9 s, followed by 12 red, green, or white letter-stimuli. The color of the stimulus determined which task the participant should perform: red letters indicated a lower/upper case decision trial, green letters a vowel/consonant decision trial, and white letters indicated a trial on which the participant should make no response (1/3 of trials). Each trial lasted 2,400 ms, with each stimulus terminated and replaced by a blank screen when a response was made or after 2,350 ms, whichever occurred first. Participants completed two single-task conditions and two dual-task conditions, a total of 6 times. Due to problems with the magnet-program interface, six participants completed only 4 blocks of the experiment and 24 completed only 5 blocks. Data for these participants were included in analyses. In all blocks, stimuli were counterbalanced so that no more than two task or no-go trials occurred in a row and no more than two upper/lower case or vowel/consonant task trials occurred in a row in dual-task blocks. Participants completed an extensive training session before scanning began. Participants were first pre-trained on the task by being shown the different color cue instructions, and practicing mapping the responses to the correct keys. Then, they received between one and three blocks of each condition (red letters or green letters) with auditory feedback indicating incorrect responses. Finally they were tested on the entire paradigm, including single and dual-task blocks, without feedback. Feedback was not provided in the experimental scanning session. In the scanner, responses were made on a LUMItouch response system (Photon Control Company) using the left and right index fingers. Task administration and collection of behavioral data were controlled using PsyScope 5X B53 (Macwhinney et al., 1997). In addition to the active task blocks, 12 additional 33.5 s resting blocks were collected in which no stimuli were presented and no response was required. These blocks were not modeled.

### 2.4. fMRI data acquisition

Multislice images of the human brain were acquired in an event-related design using a 3.0 T Philips Achieva Magnet equipped with standard quadrature head coil.

### 2.5. Anatomical MRI data

A T1-weighted scout image was acquired to determine participant position. One hundred and sixty-five contiguous 1 mm axial T1-weighted images of the whole brain were acquired for each participant with an MPRAGE sequence using the following parameters: TR 6.5 ms, TE 3 ms; flip angle 8, in-plane acquisition matrix 256 × 256 and 25.6 × 25.6 cm field of view which results in an isometric voxel size of 1 × 1 × 1 mm. All T1 scans were reviewed by a neuroradiologist for evidence of potentially clinically significant findings such as abnormal neural structure. No clinically significant findings were identified.

### 2.6. Functional MRI data

Functional data were acquired on the same Philips scanner in 6 runs, each of which included collection of 111 functional volumes using a T2*-weighted gradient-echo echo planar image sequence. Forty-one transverse slices per volume with 3.0 mm thickness and no gap in between were acquired using a field echo echo-planar imaging (FE–EPI) sequence with the following parameters: TR 2,000 ms, TE 20 ms, flip angle 72; in-plane acquisition matrix 112 × 112 matrix; which results in a voxel size 2.0 mm × 2.0 mm ×3.0 mm. Four dummy volumes were acquired at the beginning of each functional run and discarded to allow transverse magnetization immediately after radio-frequency excitation to approach its steady-state value.

## 3. Analysis

### 3.1. Demographics

First, we explored potential group related differences between the younger and older adults in terms of gender, the number of years of education, and measures of longstanding traits of intelligence including the National Adult Reading Test (NART) and the Wechsler Test of Adult Reading (WTAR) using Student’s *t*-tests.

### 3.2. Behavioral

The main variables of interest were behavioral performance for higher-load trials (i.e., switch trials) minus lower load trials (i.e., no switch trials). For accuracy, switching costs were calculated as the difference between Dual No Switch Trials and Dual Switch Trials, and mixing costs were calculated as the difference between Dual No Switch Trials and Single-task Trials. For reaction time (RT), cost was calculated in the reverse (switching costs were calculated as the difference between Dual Switch Trials and Dual No Switch Trials, and mixing costs were calculated as the difference between Single-task Trials and Dual No Switch Trials). Thus, in both cases, a larger difference score indicates a greater cost. For all participants, trials on which a response was made (correct or incorrect) in less than 300 ms were excluded, as were trials in which no response was made (time out). For RT data, only correct trials were analyzed. Both measures were analyzed using repeated and mixed models ANOVAs, and follow-up *t*-tests were used to test for cross-sectional effects of these two types of local performance costs on Age. We also explored the relation of these measures of performance costs to the two measures of intelligence, the NART and WTAR, using Pearson’s correlations.

### 3.3. fMRI pre-processing

FMRIB Software Library v5.0 (FSL) and custom-written Python code were used to perform the following pre-processing steps for each participant’s dataset: All functional images were realigned to the first volume, corrected for the order of slice acquisition, smoothed with a 5 mm^3^ non-linear kernel followed by intensity normalization, and high-pass filtered using a Gaussian kernel and cut-off frequency of 0.008 Hz. For spatial normalization, the accompanying T1-weighted high-resolution anatomic image was co-registered to the first functional volume using the mutual information co-registration algorithm implemented in FLIRT. This co-registered high-resolution image was then registered to MNI standardized space. These obtained transformation parameters were used to transfer the statistical parametric maps of the subject level analysis to standard space.

### 3.4. fMRI subject level analysis

The fMRI time-series data was pre-whitened to explicitly correct for intrinsic autocorrelations in the data. The FEAT module in FSL was used for first-level analysis. An event-related design was used to model the fMRI data, allowing us to separate timeouts (where no response was made), correct and incorrect trials, as well as switch and no switch trials. Errors and time outs were modeled together. Rest blocks were not included in the model. For all participants, a first-level analysis was run on each of their task-based runs with three regressors. The regressors represented one of the following task conditions: single-task trials, dual-task no-switch trials, and dual-task switch trials. The regressors were generated by convolving FSL’s double gamma canonical HRF with the duration of presentation of the stimulus to the participants. A second-level analysis was run on each participant by combining the first-level results for each run. At this level, two new contrasts were created from the combined first-level task regressors for each participant, reflecting activations related to (1) Local Switch Costs, calculated as dual-task switch-trials > dual-task no-switch trials, and (2) Mixing Costs, calculated as dual-task no-switch trials > single-task (no-switch) trials. In both cases, these contrasts reflected “costs” or additional activation needed to complete the more difficult task with a higher load (either sustained or transient) level as compared to an easier/lower load level task.

### 3.5. fMRI group-level analyses

After transforming each participant’s statistical parametric maps (obtained from the second-level analysis) into standard space, group-level analyses were performed using General Linear Model (GLM) with FLAME in FSL. The participant level contrasts described above were passed into the group-level analysis, dividing the sample into “younger” and “older” groups based upon their age. We first explored brain areas activated for each type of cost within each age group. Then, to explore brain areas that were age-sensitive to the different types of costs, we followed the methods used by Nashiro et al. (2018) by computing the group-level parametric maps for each of the contrasts and then using these to explore age-related over-recruitment (older > younger) and age-related under-recruitment (younger > older) for both types of costs. Voxel-wise statistical height thresholds, combined with cluster level thresholds, were employed to ensure appropriate control over false-positives. Cluster analysis, identified as voxels with a *z* value greater than 2.3 (except where noted), and that are connected to another voxel by at least a point and containing at least 20 continuous voxels, were performed on the group-level FSL FLAME results. We used a smoothness estimate of the data to implement Gaussian Random Field theory to infer the significance of each cluster (Worsley et al., 1992). Only clusters with an inferred significance of *p* < 0.01 were included in the analysis.

## 4. Results

### 4.1. Demographics

There were no significant differences between the younger and older adults groups in terms of gender [*t*(244) = 1.591, *p* = 0.113], the number of years of education [*t*(244) = −1.085, *p* = 0.279] or measures of longstanding traits of intelligence including the National Adult Reading Test (NART), [*t*(193) = −1.101, *p* = 0.272], and the Wechsler Test of Adult Reading (WTAR), [*t*(188) = −0.331, *p* = 0.741].

### 4.2. Performance by type of cost and age group

**Figure 2:**
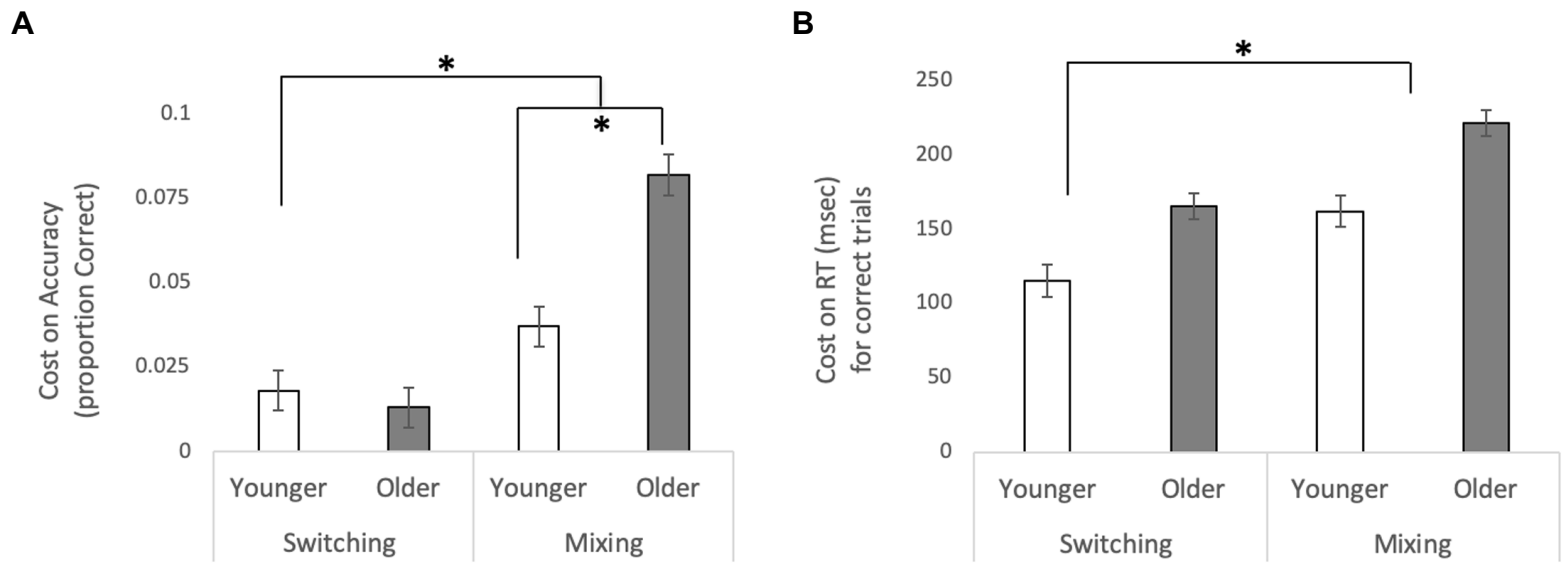
Switching and Mixing Costs for **(A)** accuracy and **(B)** reaction time for correct trials. For accuracy, switching costs were calculated as the difference between Dual No Switch Trials and Dual Switch Trials, and mixing costs were calculated as the difference between Dual No Switch Trials and Single- task Trials. For RT, cost was calculated in the reverse (switching costs were calculated as the difference between Dual Switch Trials and Dual No Switch Trials, and mixing costs were calculated as the difference between Single-task Trials and Dual No Switch Trials). In both cases, a higher value indicates a greater cost. Error bars are standard error of the mean. *p <.05.

A repeated measures ANOVA of accuracy (proportion of correct responses) with Cost (Local Switch vs. Mixing) as a within-subjects factor and Age (Younger vs. Older) as a between subjects factor revealed a main effect of Cost, *F*(1,244) = 4.081, *p* = 0.046, $\eta_{p}^{2}$ =0.016, such that performance was worse for Mixing Costs relative to Switch Costs, and a main effect of Age, *F*(1,244) = 7.184, *p* = 0.008, $\eta_{p}^{2}$ =0.029, such that overall older adults had worse performance (higher costs) overall. These effects were qualified by a significant interaction between these two factors, *F*(1, 244) = 4.976, *p* = 0.027, $\eta_{p}^{2}$ =0.02. *Post-hoc t*-tests revealed that while there was no difference in performance for Switch Costs between older and younger adults, *t*(244) = −0.531, *p* = 0.615, there was a significant difference between the two age groups for Mixing Costs, *t*(244) = 3.523, *p* = 0.001, 95% CI [0.013, 0.047], such that older adults had a significant greater cost to performance than did younger adults. These results are illustrated in Figure 2, Panel A. The analogous analyses performed on the RT data revealed a main effect of Cost *F*(1,244) = 19.834, *p* < 0.001, $\eta_{p}^{2}$ =0.075, such that at performance was worse for Mixing Costs relative to Local Switch Costs, and a main effect of Age, *F*(1,244) = 32.008, *p* < 0.001, $\eta_{p}^{2}$ =0.116, such that older adults had higher costs. The interaction between Cost and Age was not significant [*F*(1,244) = 0.164, *p* = 0.685]. These results can be visualized in Figure 2, Panel B. Mean accuracy rates and RTs for all trial types and each type of cost are presented in Table 1. Interestingly, we also found a significant correlation between WTAR and Mixing Costs for both accuracy (r = −0.206, *p* = 0.004) and reaction time (r = 0.199, *p* = 0.006), suggesting that those individuals with higher IQ show reduced costs.

**Table 1:** Mean performance values [mean (SD)] for accuracy (proportion correct) and reaction time for correct trials (ms) for the younger and older adults.

	Trial type	Younger adults		Older adults	
Accuracy	Single-task	0.961	(0.069)	0.947	(0.070)
	Dual-task no switch	0.945	(0.086)	0.901	(0.095)
	Dual-task switch	0.927	(0.091)	0.888	(0.093)
	Switching cost	0.018	(0.051)	0.013	(0.075)
	Mixing cost	0.037	(0.048)	0.082	(0.080)
Reaction time	Single-task	745.894	(104.030)	889.392	(142.439)
	Dual-task no switch	907.473	(125.817)	1110.507	(184.611)
	Dual-task switch	1022.331	(142.668)	1275.540	(222.835)
	Switching cost	114.858	(91.784)	165.033	(113.929)
	Mixing cost	161.579	(88.378)	221.115	(112.587)

### 4.3. Age-dependent brain regions related to each type of cost

#### 4.3.1. Local switch costs

**Figure 3:**
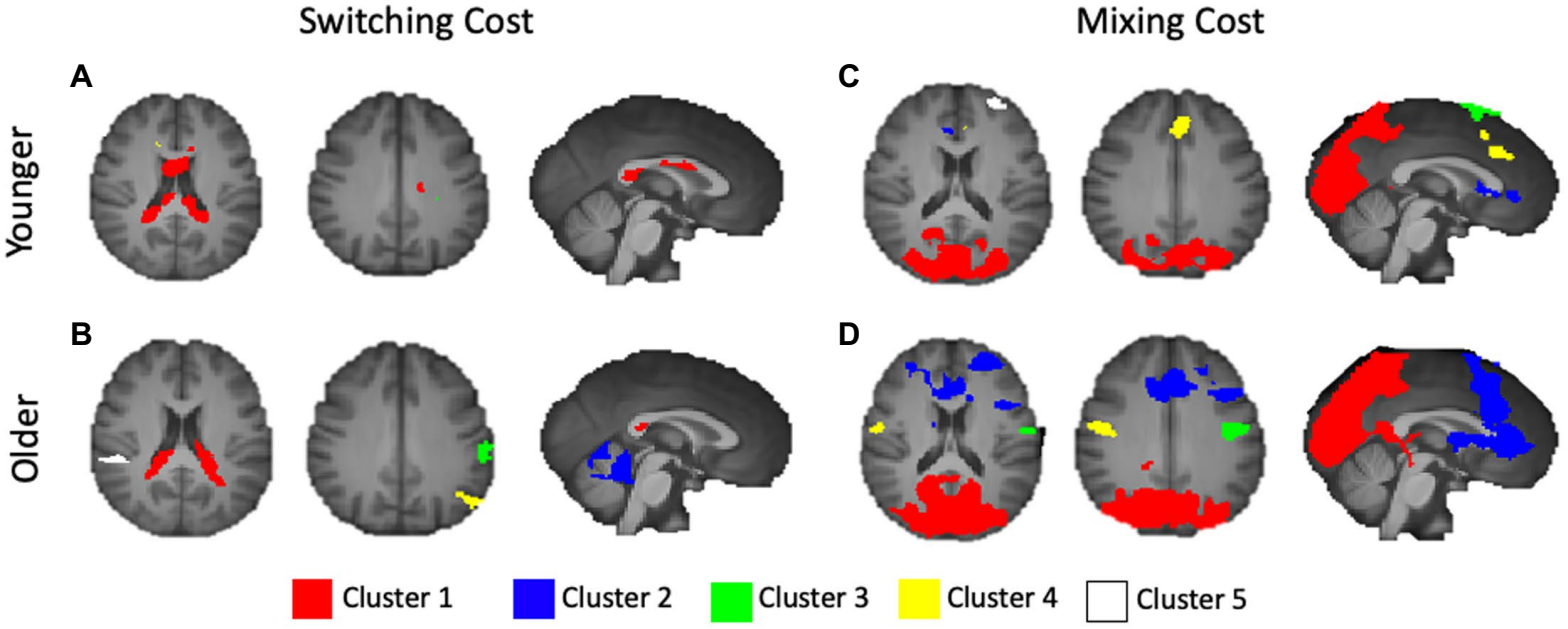
Activations for younger and older adults, separately, for switching and mixing costs. **(A)** Younger Switching Cost; **(B)** Older Switching Cost; **(C)** Younger Mixing Cost; and **(D)** Older Mixing Cost. Only the five largest clusters for each contrast are rendered. Details of each cluster are shown in Table 1, with colors representing separate clusters according to size: Cluster 1 = Red, Cluster 2 = Blue, Cluster 3 = Green, Cluster 4 = Yellow, Cluster 5 = White.

The younger adults exhibited large clusters of activation for both local switch costs and mixing costs. For local switch costs, this included a > 2,300 voxel cluster with a center of mass in the corpus callosum, which was confined largely to the limbic lobe. Several smaller areas of activation included peaks outside of defined BAs, but were nearest to frontal regions, as can be seen in Figure 3A. As can be seen in Figure 3B of the figure, older adults showed a > 1,800 voxel cluster with a peak in the corpus callosum, which bordered on the inferior parietal lobule, as well as several distinct clusters with peaks in the supramarginal gyrus (BA 40), the middle temporal gyrus (BA 21), and the pars opercularis (e.g., Broca’s area, BA 44) portion of the inferior frontal gyrus.

#### 4.3.2. Mixing costs

For mixing costs, younger adults exhibited numerous clusters of activation, including a large (>15,600 voxel) cluster of activation centered in the left secondary visual cortex (BA 18), which extended to the parietal areas. Other notable activations included an area encompassing both bilateral premotor cortex and supplementary motor area (BA 6), dorsal portions of the anterior cingulate (ACC; BA 32), and the anterior portion of BA 10, BA 44, and BA 4. The largest 5 clusters are illustrated in Figure 3C. Finally, as can be seen in Figure 3D, the older adults showed activations in several of the same areas as younger adults, including BA 4 and 6 (primary and premotor cortices), as well as large cluster, even after applying a more stringent threshold, centered in secondary visual cortex (BA 18), as well as clusters in both the dorsal part of the dorsal lateral prefrontal cortex (DLPFC, BA 9). Additional clusters of activation included centers in the frontal eye fields (BA 8), just anterior to the premotor cortex. Table 2 presents information separately for younger and older adults, and for each type of cost, and includes the associated anatomical label to which each cluster’s center of mass belongs.

**Table 2:** Clusters of activation for switching and mixing costs, separately for younger and older adults.

		Cluster	# Voxels	MAX	MAX X	MAX Y	MAX Z	Hem.	Area label (BA)
Switching costs	Young z = 2.3	1	2,361	6.62	24	−46	12	L	--
		2	14	2.91	4	−40	−10	--	--
		3	6	2.6	−26	−28	36	L	--
		4	5	2.69	14	22	18	R	--
		5	2	2.35	22	−28	28	R	--
	Old z = 2.3	1	1,891	5.83	−24	−54	12	L	--
		2	861	5.57	2	−46	−26	L	--
		3	433	3.98	−60	−30	44	L	--
		4	168	3.57	−56	−66	28	L	Angular gyrus (39)
		5	99	3.6	50	−32	18	R	Supramarginal gyrus (40)
		6	63	3.33	60	−30	40	R	Supramarginal gyrus (40)
		7	61	3.55	54	10	10	R	Pars Operc. (44)
		8	51	3.74	46	−64	32	R	Angular gyrus (39)
		9	25	3.14	10	−8	24	R	--
		10	23	2.66	52	−36	50	R	Supramarginal gyrus (40)
		11	22	3.02	50	−38	−10	R	Mid. temporal gyrus (21)
		12	20	2.83	54	12	28	R	Pars Operc. (44)
Mixing costs	Young z = 3	1	15,684	7.74	0	−82	−4	L	Sec. visual (18)
		2	1,164	4.85	−8	24	0	--	--
		3	972	4.9	−34	4	52	L	PreMotor + SuppMotor (6)
		4	286	4.1	0	30	32	L	Dorsal ACC (32)
		5	152	4.24	−30	52	14	L	Anterior PFC (10)
		6	101	3.78	36	−34	−18	R	Fusiform (37)
		7	95	4.66	−18	−22	58	L	PreMotor + SuppMotor (6)
		8	90	4.46	−62	−2	14	L	PreMotor + SuppMotor (6)
		9	62	4.22	18	14	14	R	Caudate
		10	55	3.72	22	12	52	R	PreMotor + SuppMotor (6)
		11	42	3.58	−20	−30	−2	--	--
		12	41	3.92	−54	−10	40	L	Primary motor (4)
		13	38	3.46	−46	18	30	L	Pars Operc. (44)
		14	29	3.45	26	32	32	R	Frontal eye fields (8)
		15	22	3.43	24	−24	62	R	Primary motor (4)
		16	20	3.95	22	6	68	R	PreMotor + SuppMotor (6)
	Old z = 2.3	1	22,161	10.2	−22	−74	−10	L	Sec. visual (18)
		2	13,555	7.33	0	18	2	L	--
		3	650	5.51	−48	−10	28	L	--
		4	621	6.12	48	−12	38	R	Frontal eye fields (8)
		5	53	4.67	60	−12	4	R	Primary motor (4)
		6	49	3.85	50	22	24	R	DLPFC (dorsal) (9)
		7	40	3.91	−50	−20	4	L	Primary auditory (41)
		8	37	3.49	44	20	38	R	Frontal eye fields (8)
		9	21	3.4	22	−24	56	R	--
		10	20	3.35	2	−22	62	--	PreMotor + SuppMotor (6)

All clusters had an extent of 20 or more voxels except the Younger adult Switching Cost contrast, where the largest 5 clusters are reported. ACC, Anterior Cingulate Cortex; Assoc., Association; Mid., Middle; Operc., Opercularis; PFC, Prefrontal Cortex; Supp., Supplementary; --, Outside of defined Broadman Area Mappings.

### 4.4. Age-sensitive brain regions related to each type of cost

#### 4.4.1. Switching costs

**Figure 4:**
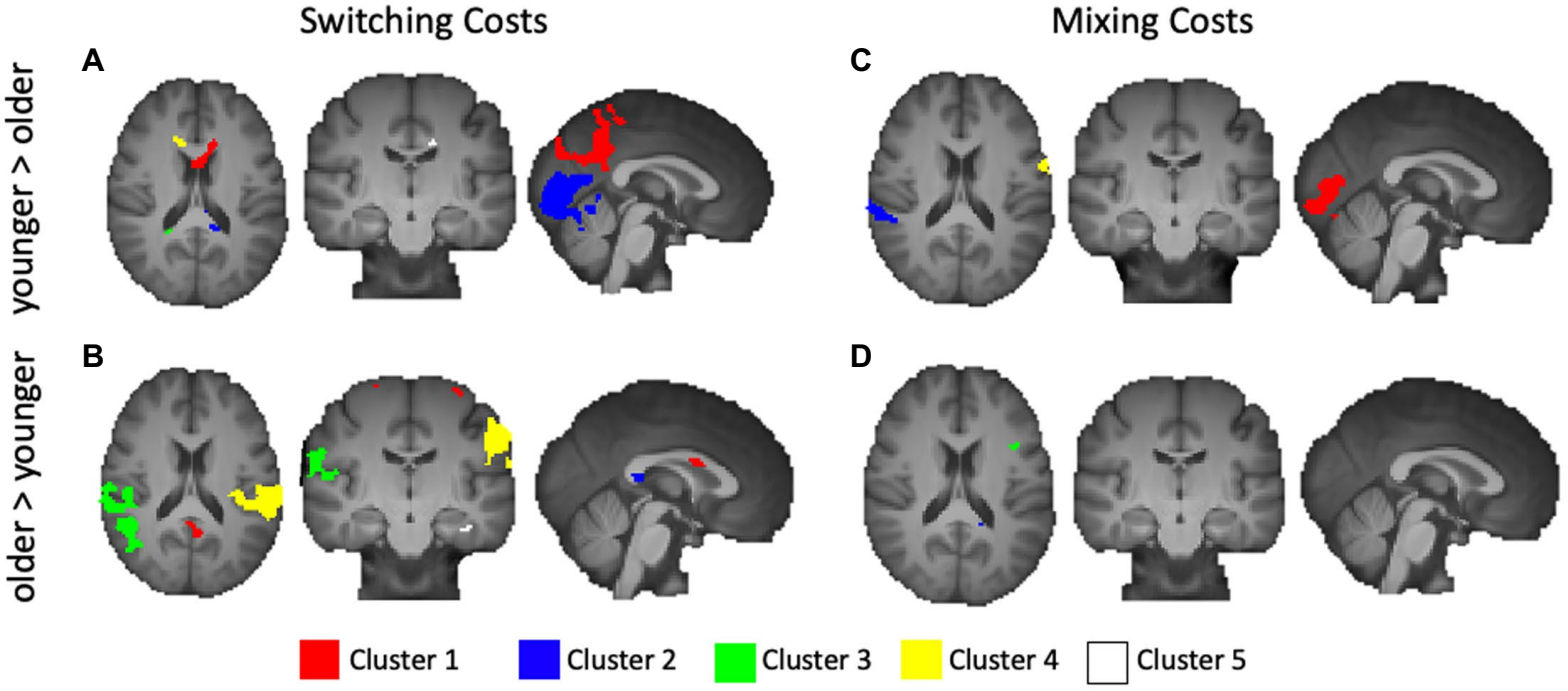
Activations from age-group level contrasts for switching and mixing costs. **(A)** Younger > Older Switching Cost; **(B)** Older > Younger Switching Cost; **(C)** Younger > Older Mixing Cost; and **(D)** Older > Younger Mixing Cost. Only the five largest clusters for each contrast are rendered. Details of each cluster are shown in Table 2, with colors representing separate clusters according to size: Cluster 1 = Red, Cluster 2 = Blue, Cluster 3 = Green, Cluster 4 = Yellow, Cluster 5 = White.

We first explored age-related under-recruitment for local switching costs by finding areas activated to a greater extent for younger adults relative to older adults. Younger adults showed numerous relatively small clusters of greater activation relative to older adults. While none of the centers of mass were within defined BAs, activations extended into the corpus callosum, frontal lobe and cingulate. The largest 5 clusters are rendered in Figure 4A, and described in Table 3.

**Table 3:** Local switching cost.

	Cluster	# Voxels	MAX	MAX X	MAX Y	MAX Z	COG X	COG Y	COG Z	Hem.	Area label (BA)
Young > old	1	221	3.93	−12	20	20	−0.4	7.04	21	--	--
	2	143	3.34	−14	−44	16	−5.9	−37	15	L	--
	3	53	3.34	24	−46	12	22.9	−45	13	R	--
	4	45	3.47	14	22	18	14.6	22.8	18	R	--
	5	38	3.13	−12	−14	30	−14	−15	30	L	--
	6	33	3.1	−34	2	18	−35	2.36	17	L	--
	7	23	2.78	36	−42	0	34.8	−42	−1	R	--
Old > young	1	3,102	4.08	−24	−44	62	3.37	−44	51	R	Visual motor cortex (7)
	2	2,950	3.88	0	−84	−12	3.26	−74	−4	--	--
	3	1,983	4.15	48	−34	10	54.3	−40	21	R	Sup. temporal gyrus (22)
	4	1,441	4.53	−56	−18	36	−56	−26	25	L	Primary sensory (1)
	5	971	4.86	−32	−40	−14	−36	−42	−17	L	--
	6	818	4.06	28	38	44	16.5	41.7	34	R	DLPFC (dorsal) (9)
	7	424	3.76	−44	−48	18	−49	−57	17	L	--
	8	387	4.05	36	−30	−18	29.1	−33	−12	R	Parahippocampal gyrus (36)
	9	173	3.53	42	2	−8	45.6	−1.7	−8	R	Insula (BA 13)
	10	116	3	−42	−58	38	−47	−62	33	L	Angular gyrus (39)
	11	113	3.01	34	−80	−8	30.8	−80	−7	R	Visual Assoc. (38)
	12	97	3.04	−60	4	14	−57	0.37	9.9	L	Pars Operc. (44)
	13	89	3.21	18	−40	−22	18.9	−42	−25	--	--
	14	82	2.93	−14	−36	46	−13	−35	43	L	Dorsal PCC (31)
	15	72	2.92	0	28	30	−0.7	22.3	32	L	Dorsal ACC (32)
	16	71	2.97	−10	66	16	0.82	62.9	14	L	Anterior PFC (10)
	17	67	3.1	54	10	8	54.8	8.9	6.9	R	Pars Operc. (44)
	18	63	2.89	−26	−24	52	−30	−25	50	--	--
	19	61	3.3	−12	−10	−20	−16	−7.1	−16	L	--
	20	56	3.02	6	−4	50	3.89	−4.3	51	R	PreMotor + SuppMotor (6)
	21	49	3.17	2	24	66	0.24	29.2	62	--	--
	22	44	2.98	2	−20	44	2.87	−22	46	R	Ventral ACC (24)
	23	42	3.12	38	−12	8	37.3	−11	8.6	R	Insula (13)
	24	36	3.11	2	−12	70	1.73	−14	69	--	--
	25	30	2.7	2	−6	40	1.73	−7.3	40	R	Ventral ACC (24)
	26	29	2.7	38	−18	46	39.6	−17	46	R	Primary motor (4)
	27	28	2.77	−22	−66	−6	−24	−66	−7	L	Visual Assoc. (19)
	28	27	3.27	−60	−20	−10	−57	−17	−10	L	Mid. temporal gyrus (21)
	29	26	2.71	−40	−64	−24	−39	−65	−22	L	Cerebellum
	30	24	3	2	26	18	0.93	26.6	18	L	Ventral ACC (24)
	31	24	2.65	−34	−76	−20	−33	−77	−20	L	Cerebellum

Clusters of activation with an extent of 20 or more voxels, thresholded at z = 2.3. ACC, Anterior Cingulate Cortex; Assoc., Association; BA, Broadman Area; Mid., Middle; Operc., Opercularis; PCC, Posterior Cingulate Cortex; PFC, Prefrontal Cortex; Sup., Superior; Supp., Supplementary; --, Outside of defined Broadman Area Mappings.

We then explored age-related over-recruitment through a contrast of old>young. Whereas there were relatively few areas activated to a greater extent by younger relative to older adults, our results indicated numerous distinct areas, shown in Table 3, that older adults activated to a greater extent than younger adults. Figure 4B presents the largest 5 of these clusters, which included peaks in the dorsal aspect of the DLPFC (BA 9), primary somatosensory cortex (BA 1), superior temporal gyrus (BA 22), left lateralized dorsal posterior and anterior cingulate (BA 31 and 32), right ventral ACC (BA 24), BA 6, 4, the middle temporal gyrus (BA 21), and the pars opercularis.

#### 4.4.2. Mixing costs

We performed the same series of analysis for mixing costs, first exploring areas that showed age-related under recruitment, and then exploring areas that showed age-related over recruitment. For the contrast of younger > older, the largest cluster centered in the secondary visual cortex (BA 18). Other areas included right lateralized superior temporal gyrus (BA 22), the supramarginal gyrus (BA 40), bilateral fusiform gyrus (BA 37), BA 6, and a relatively small cluster centered in the parahippocampal gyrus. These results are illustrated in Figure 4C. The older adults showed a greater magnitude of activation relative to younger adults for mixing costs in several prefrontal regions including the right anterior PFC (BA 10) and the left pars opercularis (BA 44, shown in green in Figure 4D). An additional small cluster was noted in the middle temporal gyrus (BA 21), along with another cluster whose center of mass was outside of defined BA, but bordered the precentral gyrus. Table 4 presents information for age related under and over recruitments for mixing costs.

**Table 4:** Mixing Cost.

Contrast	Cluster	# Voxels	MAX	MAX X	MAX Y	MAX Z	COG X	COG Y	COG Z	Hem.	Area label (BA)
Young > old	1	1,478	3.75	−18	−92	−18	7.63	−87	−3	R	Visual Assoc. (18)
	2	282	3.45	64	−32	18	59.8	−35	20	R	Sup. temporal gyrus (22)
	3	177	3.61	48	−2	−10	44.5	−1.1	−9	R	Sup. temporal gyrus (22)
	4	102	4	−60	0	14	−61	0.82	16	L	PreMotor + Supp. Motor (6)
	5	79	3.02	28	−58	−14	30.8	−59	−16	R	Fusiform (37)
	6	76	2.87	−30	−42	−18	−36	−42	−21	L	Fusiform (37)
	7	62	2.92	−62	−28	16	−62	−27	15	L	Supramarginal gyrus (40)
	8	57	2.86	30	−38	−24	33.3	−42	−23	R	Fusiform (37)
	9	54	2.86	−32	−56	−22	−30	−54	−22	L	--
	10	49	3.01	6	−56	0	6.67	−55	−3	R	--
	11	48	3.07	−16	−24	56	−17	−23	57	L	--
	12	43	3.23	56	10	8	56.8	9.83	8.9	R	Pars Operc. (44)
	13	43	2.73	−6	−54	−4	−7	−53	−3	L	--
	14	41	2.78	−34	−10	68	−28	−8.1	64	L	--
	15	41	2.76	−42	−34	18	−38	−30	17	L	Supramarginal gyrus (40)
	16	37	3.01	−28	−8	−8	−27	−6.4	−7	L	--
	17	34	3.32	24	46	44	23.1	45	44	R	Frontal eye fields (8)
	18	26	3.05	18	14	14	17.5	14.1	14	R	Caudate
	19	25	2.89	20	−32	−6	21.7	−33	−4	R	Parahippocampal gyrus (36)
	20	24	3.18	2	24	66	1.76	21.8	66	--	--
	21	24	2.67	−38	−90	−14	−36	−91	−11	L	Secondary visual (18)
	22	22	2.9	46	16	−10	41.6	13.2	−11	R	--
	23	20	3.14	−42	−76	−20	−41	−74	−19	L	--
Old > young	1	76	3.09	32	46	−2	28.9	44.4	−6	R	Anterior PFC (10)
	2	67	2.95	−16	−46	12	−20	−47	12	L	--
	3	65	3.22	−40	12	16	−39	12.4	14	L	Pars Operc. (44)
	4	39	3.03	20	−46	14	20.6	−45	12	R	--
	5	33	2.92	−44	−6	26	−44	−7	27	L	--
	6	27	3.36	60	−28	4	58.2	−27	3.6	R	Mid. temporal gyrus (21)
	7	24	2.78	−8	20	22	−8	19.2	24	L	--

Clusters of activation with an extent of 20 or more voxels, thresholded at z = 2.3. ACC, Anterior Cingulate Cortex; Assoc., Association; BA, Broadman Area; Mid., Middle; Operc., Opercularis; PCC, Posterior Cingulate Cortex; PFC, Prefrontal Cortex; Sup., Superior; Supp., Supplementary; -- = Outside of defined Broadman Area Mappings.

## 5. Discussion

In the current study, we used fMRI to examine the effect of age on two different costs that arise from task switching: local switch costs, measured as the difference between switch and no switch trials within dual-task blocks, and mixing costs, measured as the difference between no switch trials in dual-task, as opposed to single-task trials. We replicated previous behavioral work showing age-related differences in accuracy-based mixing costs, but not switching costs, with the older adults exhibiting strong mixing costs, but only modest local switch costs relative to younger adults. We further found that while younger and older adults recruited similar brain areas for local switching costs, older adults also showed additional areas of activation, primarily in a fronto-parietal network that included the inferior frontal gyrus (pars opercularis), angular gyrus and supramarginal gyrus, as well as the middle temporal gyrus, all areas associated with complex language functions. For mixing costs, on the other hand, we found that older adults under-recruited a number of areas relative to younger adults. These under recruitments were located in frontal, parietal and temporal areas, including the pars opercularis, pre and post motor cortices, the superior temporal gyrus (BA 22), and the fusiform gyrus.

Local switching costs may involve transient cognitive processes needed on a trial by trial basis to resolve interference from the alternative task (Monsell, 2003; Jonides and Nee, 2005, 2006; Philipp et al., 2008; Kiesel et al., 2010), or reconfigure the new task-set by upregulating attentional resources toward the currently relevant task-set and adopt the correct stimulus–response mapping (Kray and Lindenberger, 2000; Monsell, 2003; Koch et al., 2005). Both the lack of age-related differences for local switch costs and the finding of additional neural activation on the part of the older adults relative to younger adults suggest that older adults may be able to engage compensatory neural circuits to successfully achieve these trial-level transient shifts.

Mixing costs, on the other hand, may reflect the need to keep different amounts of information in working memory, or the need for sustained use of inhibitory processes to suppress the non-cued task rules. For working memory, the argument is straightforward: the dual-task block constitutes a higher working memory load than the single-task blocks, as it is only in the dual-task block that two decision rules need to be kept in mind at all times, regardless of whether the particular trial itself is a switch or a non-switch trial. Thus, a lower working memory capacity should lead to greater mixing, but not local switch costs, as the working memory load would be equivalent across switch and no-switch trials within a dual-task block. To test this hypothesis, Keele and Rafal (2000) gave left prefrontal patients a task switching paradigm in which both working memory load and sequence demands were equated across single and dual-task blocks (Keele and Rafal, 2000). In this case, unlike in “standard” task switching paradigms, if working memory were driving differences between local and mixing costs, one would expect to greatly reduce or even eliminate the mixing cost effect. However, they found that the patients still exhibited large mixing costs, but not local switch costs, a finding which suggests that working memory load differences across single and dual-task trials are not a mechanistic explanation for differences between local and mixing costs.

For inhibition, the story is more complex. Inhibitory processes have been argued to be a critical component of task switching, as they allow the non-relevant task-set to be suppressed so that the appropriate action for the relevant, cued task-set, can be acted upon. Mayr and Keele (2000) provided evidence for this idea by administering a lag-2-repetition paradigm to compare performance on Task A in an ABA vs. a CBA task trial sequence. According to a priming or heightened attention view, performance on the second instance of Task A should be facilitated in the ABA sequence as compared to the CBA sequence, as task A should be primed and highly activated due to its occurrence two trials before. However, this was not what they found. Instead, performance of Task A was worse following the AB sequence as compared to the CB sequence. The fact that the second instance of Task A suffers in the ABA sequence points to a role for inhibition in task switching: much like the findings from negative priming studies reported by Tipper (1985), the rules for Task A are inhibited when Task A is first encountered as a distractor (e.g., on the B trial), and have not been released from inhibition by the time the second instance of that Task A occurs, which leads to a decrement in performance (longer RTs, more errors). In the case of local switching costs, because participants must consistently switch tasks in the dual-task block, and because the effects of inhibiting the task rules carry over across multiple trials, the effects of inhibition on no switch vs. task switch trials within a dual-task block are presumably equally present across the whole dual-task block. Thus, failures of inhibitory ability should not manifest in local switching costs. The predictions are different for mixing costs, however. In the single-task condition, there is no need to inhibit the second task (as there is no second task), and thus inhibitory processes aren’t required at all in the case of single task blocks. No switch trials within dual-task blocks, on the other hand, may be affected by the sustained need for inhibitory abilities across the entire block. Mixing costs may therefore reflect a difference between one condition in which inhibitory processes are required (dual-task no switch trials) and one condition in which they are not needed (single-task trials). Studies that show age-related differences in global switch costs (when comparing dual task to single task blocks), may thus be picking up on this difference. Numerous behavioral and imaging studies have revealed a pronounced age-related inhibitory deficit (Dempster, 1992; Anderson and Spellman, 1995; Radvansky et al., 2005; Eich et al., 2017; Siegel and Eich, 2021). In the current study, the fact that older adults show both a behavioral deficit when comparing dual-task no switch trials and single-task trials, coupled with the fact that, unlike in the local switching cost contrast which presumably does not index inhibition, older adults show relatively few compensatory activations for mixing costs, raises the possibility that the deficits derive from older adult’s inability to effectively sustain inhibitory processes needed to successfully handle the no switch trials across the dual-task context.

The results presented here suggest that older adults do not have difficulty with the actual switching component of task switching. Indeed, the local switch cost variable was derived from switch and no switch trials within the dual-task block, and yet was age invariant. On the other hand, the mixing cost, which was derived from no-switch trials across the single task and dual task blocks was significantly related to age. Interestingly, however, older adults did show recruitment of additional brain areas for local switch costs, suggesting that compensatory processes may be buffering against performance related deficits. As deficits in executive control have predicted subsequent global cognitive decline (Clark et al., 2012) or discriminated patients with early stage Alzheimer’s disease from healthy older adults (Hutchison et al., 2010), by distinguishing those executive control processes that are uniquely sensitive to age or age-related disease from those that are age or disease invariant, assessments and interventions can be made more precise.

## Data availability statement

The raw data supporting the conclusions of this article will be made available by the authors, without undue reservation.

## Ethics statement

The studies involving human participants were reviewed and approved by Columbia University Medical Center AAAB0596. The patients/participants provided their written informed consent to participate in this study.

## Author contributions

TE, YG, CL, and JS analyzed the data. TE, CL and JS created the figures. TE, YG, CH, and YS wrote the paper. All authors contributed to the article and approved the submitted version.

## Funding

This work was supported by the National Institutes of Health, National Institute on Aging Grant R01AG026158.

## Conflict of interest

The authors declare that the research was conducted in the absence of any commercial or financial relationships that could be construed as a potential conflict of interest.

## Publisher’s note

All claims expressed in this article are solely those of the authors and do not necessarily represent those of their affiliated organizations, or those of the publisher, the editors and the reviewers. Any product that may be evaluated in this article, or claim that may be made by its manufacturer, is not guaranteed or endorsed by the publisher.

## Author’s disclaimer

Any opinions, findings, and conclusions or recommendations expressed in this material are those of the authors and do not necessarily reflect the views of the NIH/NIA.
